# XVII Reunión Post-ECTRIMS: Revisión de las Novedades Presentadas en el Congreso ECTRIMS 2024 (Parte II)

**DOI:** 10.31083/RN39228

**Published:** 2026-01-21

**Authors:** Óscar Fernández, Adrián Arés, Eduardo Agüera, Yolanda Aladro, Ana Alonso, Rafael Arroyo, Luis Brieva, Carmen Calles, Ana Belén Caminero, Tamara Castillo-Triviño, Lucienne Costa-Frossard, Sara Eichau, Miguel Ángel Hernández, Lamberto Landete, Miguel Llaneza, Sara Llufriu, José E. Meca-Lallana, Virginia Meca-Lallana, Ester Moral, Celia Oreja-Guevara, José María Prieto, Lucía Romero-Pinel, Andreu Vilaseca, Alfredo Rodríguez-Antigüedad

**Affiliations:** ^1^Departamento de Farmacología, Facultad de Medicina; Instituto de Investigación Biomédica de Málaga (IBIMA), Hospital Universitario Regional de Málaga, Universidad de Málaga, 29010 Málaga, España; ^2^Complejo Asistencial Universitario de León, 24008 León, España; ^3^Servicio de Neurología, Hospital Reina Sofía, 14004 Córdoba, España; ^4^Servicio de Neurología, Hospital Universitario de Getafe, 28905 Madrid, España; ^5^Unidad de Esclerosis Múltiple, Servicio de Neurología, Hospital Regional Universitario de Málaga, 29010 Málaga, España; ^6^Servicio de Neurología, Hospital Universitario Quirónsalud, 28223 Madrid, España; ^7^Departamento de Medicina, Universitat de Lleida, Hospital Universitari Arnau de Vilanova, 25198 Lleida, España; ^8^Servicio de Neurología, Hospital Universitario Son Espases, 07120 Palma de Mallorca, España; ^9^Departamento de Neurología, Complejo Asistencial de Ávila, 05071 Ávila, España; ^10^Servicio de Neurología, Hospital Universitario Donostia, Grupo de Neuroinmunología, IIS Biogipuzkoa, 20014 Donostia, España; ^11^CSUR de Esclerosis Múltiple, Hospital Ramón y Cajal, 28034 Madrid, España; ^12^Servicio de Neurología, Hospital Universitario Virgen Macarena, 41009 Sevilla, España; ^13^Servicio de Neurología, Hospital Nuestra Señora de Candelaria, 38010 Santa Cruz de Tenerife, España; ^14^Servicio de Neurología, Hospital Universitario Doctor Peset, 46017 Valencia, España; ^15^Servicio de Neurología, Hospital Universitario Central de Asturias, 33011 Oviedo, España; ^16^Unidad de Neuroinmunología y Esclerosis Múltiple, Hospital Clínic de Barcelona e IDIBAPS, 08036 Barcelona, España; ^17^Unidad de Neuroinmunología Clínica y CSUR Esclerosis Múltiple, Servicio de Neurología, Hospital Clínico Universitario Virgen de la Arrixaca (IMIB-Arrixaca), Cátedra de Neuroinmunología Clínica y Esclerosis Múltiple, Universidad Católica San Antonio (UCAM), 30120 Murcia, España; ^18^Servicio de Neurología, Hospital Universitario de la Princesa, 28006 Madrid, España; ^19^Servicio de Neurología, Complejo Hospitalario Universitario Moisès Broggi, 08980 Barcelona, España; ^20^Servicio de Neurología, Hospital Clínico San Carlos, IdISSC, Madrid; Departamento de Medicina, Facultad de Medicina, Universidad Complutense de Madrid (UCM), 28040 Madrid, España; ^21^Servicio de Neurología, Instituto de Investigación Sanitaria de Santiago de Compostela (IDIS), 15706 Santiago de Compostela, España; ^22^Departamento de Neurologia, Hospital Universitari de Bellvitge-IDIBELL, 08908 L’Hospitalet de Llobregat, España; ^23^Servei de Neurologia, Hospital Universitario Vall d’Hebron, CEMCAT, 08035 Barcelona, España; ^24^Servicio de Neurología, Hospital Universitario Cruces, 48903 Barakaldo, España

**Keywords:** ECTRIMS, esclerosis multiple, post-ECTRIMS, ECTRIMS, multiple sclerosis, post-ECTRIMS

## Abstract

**Introducción:**

La XVII edición de la reunión post-Comité Europeo para el Tratamiento y la Investigación de la Esclerosis Múltiple (ECTRIMS) se celebró los días 4 y 5 de octubre de 2024 en Madrid. Este evento reunió a neurólogos especializados en esclerosis múltiple (EM) en España, quienes presentaron un resumen de los avances más relevantes discutidos en el congreso ECTRIMS, celebrado días antes en Copenhague.

**Objetivo:**

Sintetizar las principales novedades en investigación clínica sobre EM.

**Desarrollo y Conclusiones:**

Durante la reunión, se analizaron los cambios inmunológicos a lo largo de la vida y en respuesta a los tratamientos para la EM. También se discutió el impacto del envejecimiento y se revisaron las novedades relacionadas con los tratamientos sintomáticos, el ejercicio físico, la fatiga y los trastornos del sueño. Se destacó la importancia de abordar los retos pendientes en la medición del fallo terapéutico y el manejo de los tratamientos para la EM secundariamente progresiva. Además, se presentaron datos de registros y estudios de vida real, incluidos nuevos análisis sobre cladribina, subrayando la necesidad de recopilar información de seguridad. En relación con las terapias de reconstitución inmune, se exploraron los mecanismos de acción y el proceso de reconstitución del sistema inmune tras el tratamiento. Por último, entre las innovaciones terapéuticas, se reportaron los primeros resultados de los estudios con terapia del inglés chimeric antigen receptor T-cell (CAR-T) en EM, que representan un avance prometedor en el manejo de la enfermedad.

## Abstract (en)

**Introduction:**

The XVII edition of the post-European Committee for Treatment and Research in Multiple Sclerosis (ECTRIMS) meeting was held on 4–5 October 2024 in Madrid. This event was attended by Spanish neurologists specialized in multiple sclerosis (MS), who presented a summary of the most relevant advances discussed at the ECTRIMS congress, held days before in Copenhagen.

**Aim:**

To summarize the main new findings in clinical research on MS.

**Content and Conclusions:**

The immunological changes throughout life and in response to MS treatments were analyzed. The impact of aging was also discussed, along with new developments related to symptomatic treatments, physical exercise, fatigue, and sleep disorders. Emphasis was placed on addressing ongoing challenges in measuring therapeutic failure and managing treatments for secondary progressive MS. Additionally, data from registries and real-world studies were presented, including new analyses on cladribine, highlighting the need to collect safety information. Regarding immune reconstitution therapies, mechanisms of action and the immune system’s reconstitution process after treatment were explored. Finally, regarding therapeutic innovations, the first results from chimeric antigen receptor (CAR)-T cell therapy studies in MS were reported, representing a promising advancement in disease management.

## 1. Introducción

Los días 4 y 5 de octubre de 2024 se celebró en Madrid la XVII edición de la reunión post-Comité Europeo para el Tratamiento y la Investigación de la Esclerosis Múltiple (ECTRIMS). Como cada año, neurólogos expertos en esclerosis múltiple (EM) en España resumieron las principales novedades del congreso ECTRIMS celebrado en Copenhagen unos días antes. El presente artículo describe las novedades relacionadas con el tratamiento y con los cambios clínicos a lo largo de la enfermedad.

Las ponencias seleccionadas abarcan desde los aspectos fisiopatológicos y diagnósticos hasta el manejo terapéutico y el seguimiento clínico de las personas con EM. La selección de ponencias se realizó siguiendo un enfoque temático coherente con el utilizado en ediciones previas de las jornadas Post-ECTRIMS y en los artículos publicados desde su inicio, con el objetivo de garantizar una cobertura equilibrada y continuada de las líneas de investigación con mayor impacto potencial en la práctica clínica.

## 2. Cambios Inmunológicos Durante la Vida y el Tratamiento en la EM

### 2.1 Cambios en el Sistema Inmunitario 

A lo largo de la vida, el sistema inmune experimenta cambios, como la disminución de la tolerancia inmune, que contribuye al desarrollo de enfermedades autoinmunes [1]. Los cambios hormonales también desempeñan un papel importante en estas alteraciones inmunológicas. Por ejemplo, durante el embarazo, el aumento de los niveles de estrógenos y progesterona favorece una respuesta inmune antiinflamatoria.

Con el envejecimiento del sistema inmune, conocido como inmunosenescencia, se observa un aumento en la susceptibilidad a infecciones y la proliferación de células cancerígenas, acompañado de una menor eficacia en la respuesta a las vacunas. En los pacientes con EM hay inmunosenescencia prematura, además de una mayor progresión de la discapacidad y una respuesta diferente a los tratamientos modificadores de la enfermedad (TME) [1]. Un estudio reciente en pacientes mayores de 55 años mostró que el tiempo hasta el diagnóstico en estos pacientes es casi el doble que en pacientes más jóvenes, probablemente influido por un mayor número de comorbilidades. El número de comorbilidades, signos piramidales y cerebelosos, y un mayor número de brotes fueron factores de peor pronóstico en los pacientes mayores [2].

### 2.2 Estrategias de Tratamiento en Pacientes Pediátricos

Aunque los pacientes con EM de inicio pediátrico tardan más en desarrollar discapacidad, lo hacen a una edad más temprana [3]. Diferentes estudios han demostrado que estos pacientes se benefician de los TME, llevando a la aprobación de fingolimod (≥10 años, en pacientes con alta actividad), teriflunomida (≥10 años), dimetilfumarato (DMF, ≥13 años), e interferón-beta-1a (≥12 años) en Europa y natalizumab (≥12 años, en pacientes con alta actividad) en Italia [3]. Además, como ocurre con los adultos, el uso temprano de tratamiento modificador de la enfermedad (TME) de alta eficacia minimiza el riesgo de acúmulo de discapacidad a edades tempranas así como de deterioro cognitivo [4].

### 2.3 Fecundación In Vitro, Embarazo y Lactancia

Contrariamente a lo que se pensaba hace años, las técnicas de reproducción asistida no aumentan el riesgo de brotes, por lo que se pueden realizar en pacientes con EM [5, 6]. En mujeres embarazadas, Hellwig recomendó continuar con natalizumab hasta la semana 30–34 [6]. Los antagonistas del receptor de esfingosina-1-fosfato (S1P) deberían evitarse ya antes de la concepción, debido al riesgo de malformaciones, entre otros. Idealmente, se debería planificar un cambio de TME antes del embarazo a otros tratamientos como cladribina, terapias anti-CD20 o natalizumab [6]. En un estudio de 336 embarazos en mujeres expuestas a cladribina antes del embarazo o durante el primer trimestre con 157 resultados conocidos solo se ha visto un caso de malformación, aunque se recomienda mantener la contracepción durante 6 meses tras la última dosis [6]. Otros estudios muestran que el DMF y los anti-CD20 se podría mantener hasta antes del embarazo [6, 7].

Respecto a cuándo retomar el TME después de la lactancia, se recomienda empezar lo antes posible, ya que el control de la actividad es mejor que con la lactancia en exclusiva [5, 6].

### 2.4 Menopausia

Los síntomas de la EM pueden solaparse con los de la menopausia. Los tratamientos para este periodo incluyen los farmacológicos (fezolinetant, indicado para los sofocos), y los no farmacológicos (terapia cognitivo-conductual e hipnosis) [8, 9]. La menopausia puede desencadenar una mayor neurodegeneración, aunque es difícil de medir debido a la dificultad de capturar cambios longitudinales específicos de la menopausia y de ajustar factores confusores como la edad y la variabilidad individual [8].

### 2.5 Disfunción Sexual

Un alto porcentaje de mujeres (62,5%) y hombres (66%) con EM tienen disfunción sexual [10, 11]. Sin embargo, esta disfunción está poco reconocida, probablemente debido a que no se suele preguntar por ello en consulta, aunque a la mayoría de los pacientes les gustaría que les preguntaran [12]. Se recomendó preguntar por la disfunción sexual de manera activa [13].

## 3. Envejecimiento con EM: Implicaciones Para el Tratamiento

### 3.1 Biomarcadores y Mecanismos de la Enfermedad

Cada vez hay más pacientes con EM de edad avanzada y que debutan más tarde, estos últimos con mayor probabilidad de comenzar con formas progresivas, y de alcanzar una mayor discapacidad y deterioro cognitivo en menos tiempo [14, 15]. La discapacidad y deterioro cognitivo se asocian con un mayor brain-del inglés brain predicted age difference (PAD) (diferencia entre la edad cerebral prevista y la cronológica) en pacientes con EM [16]. Esta diferencia es aún mayor en pacientes con EM secundariamente progresiva (EMSP) y en aquellos con mayor duración de la enfermedad.

Algunas estrategias para ralentizar la edad biológica son los hábitos de vida saludables, los antioxidantes, y los medicamentos para la hipertensión, diabetes o hipercolesterolemia [17]. Los marcadores somáticos de envejecimiento, como la longitud de los telómeros de los leucocitos, o de edad reproductiva pueden ayudar también a entender la progresión.

Por otra parte, se ha propuesto un modelo de organoides para la evaluación de TME para la neurodegeneración [18]. El estudio exploró el rol de las células gliales en la inflamación y progresión, utilizando organoides cerebrales generados a partir de células madre pluripotentes humanas, y mostró cómo los mediadores solubles en el líquido cefalorraquídeo (LCR) condujeron a la muerte de oligodendrocitos y a la neurodegeneración inflamatoria.

### 3.2 Estrategias de Tratamiento

**Fig. 1.**
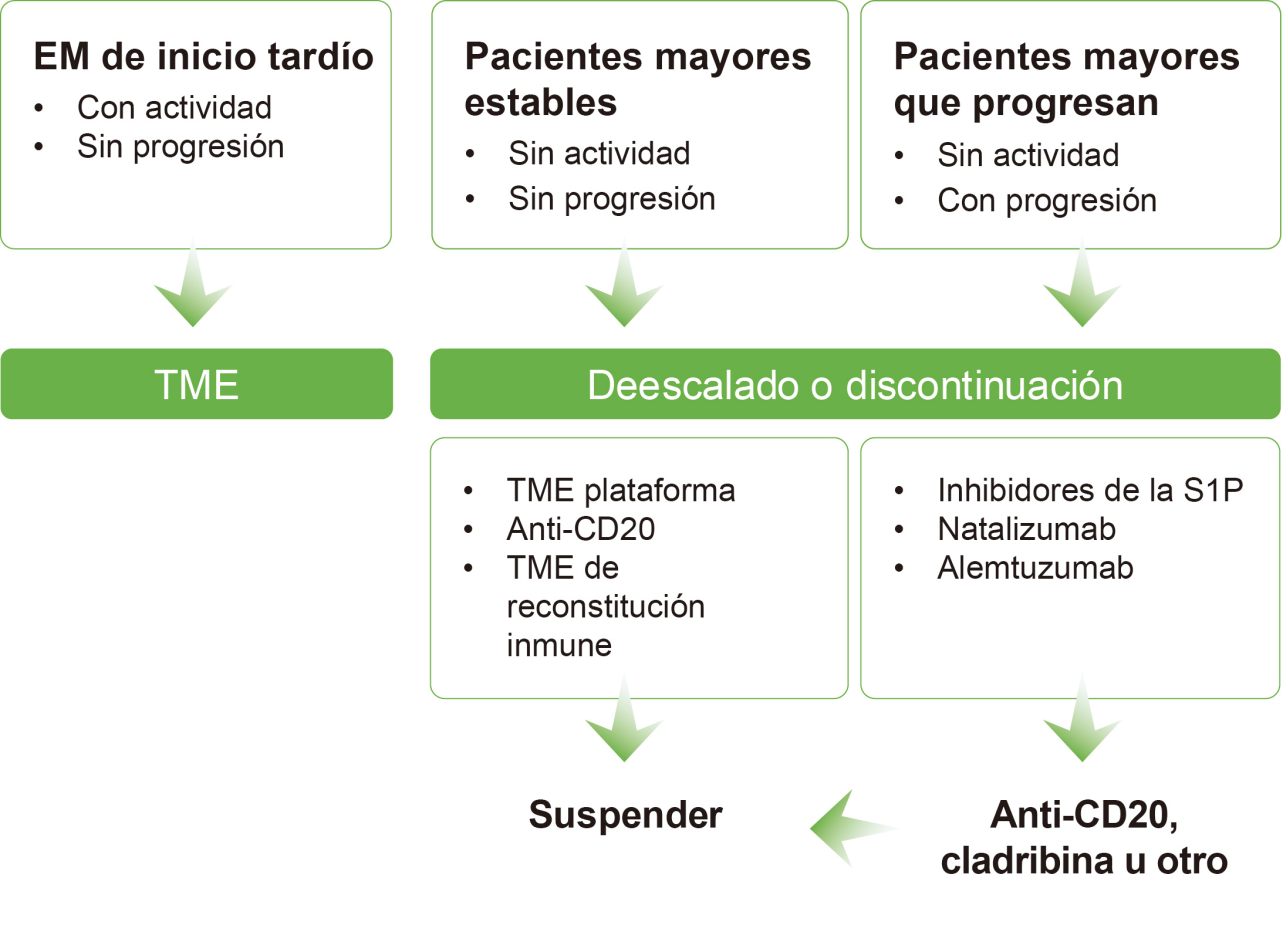
**Estrategia terapéutica potencial en pacientes de edad avanzada**. EM, esclerosis múltiple; S1P, esfingosina 1-fosfato; TME, tratamiento modificador de la enfermedad. Esta figura fue creada utilizando la versión oficial de Adobe Illustrator 2024 (Adobe Inc., San José, CA, EE. UU.).

Dado que con el tiempo la actividad inflamatoria disminuye y la progresión de la discapacidad aumenta, es importante tener en cuenta el riesgo y beneficio de los tratamientos en esta población. El estudio del registro de pacientes sueco mostró que en pacientes tratados con anti-CD20 el riesgo de infección es acumulativo a lo largo del tiempo [19]. Además, en pacientes tratados con natalizumab, el riesgo de leucoencefalopatía multifocal progresiva (PML) es mayor a partir de los 50 años y el riesgo de linfopenias relacionadas con dimetil fumarato aumenta a partir de los 55 años [20]. Las recomendaciones de tratamiento expuestas se presentan en la Fig. 1.

Un estudio comparó el efecto de discontinuar TME de alta eficacia en la actividad de la enfermedad (brotes y lesiones en resonancia magnética [RM]) en pacientes con EM recurrente remitente (EMRR) o EMSP sin brotes en los dos años antes del estudio [21]. Los pacientes tratados con natalizumab o fingolimod tuvieron mayor riesgo de presentar actividad que los tratados con anti-CD20. La edad fue un factor protector, decreciendo un 7% el riesgo de presentar actividad con cada año de edad. Con estos resultados, recomendaron cambiar a los pacientes tratados con fingolimod o natalizumab a anti-CD20, a cladribina, u a otro TME de moderada eficacia [21].

## 4. Tratamiento Sintomático

### 4.1 Dolor Neuropático y Neuralgia del Trigémino

El dolor es un síntoma prevalente en pacientes con EM (29–86%). El tratamiento farmacológico para el dolor neuropático, como dolor en las extremidades, fenómeno de Lhermitte y neuralgia del trigémino, cuenta con opciones respaldadas por evidencia de clase 1. Estos medicamentos son principalmente los anticonvulsivos, antidepresivos y relajantes musculares más antiguos (Tabla 1, Ref. [22, 23]). Además, hay enfoques no farmacológicos efectivos para el manejo del dolor neuropático, como la psicoterapia, las terapias de estimulación neuromoduladora no invasiva o la actividad física [22].

**Tabla 1.** **Tratamiento farmacológico para el dolor neuropático y neuralgia del trigémino**.

Dolor neuropático	
Clase	Tratamiento
Cannabinoides	Oral, mucosa, fumado, vaporizado
Relajantes musculares	Tizanidina^1^, baclofeno^1^, dantroleno
Anticonvulsivos	Carbamazepina^1^, lamotrigina^1^, gabapentina, pregabalina, fenitoína, benzodiacepinas
Antidepresivos	Antidepresivos tricíclicos (amitriptilina)^1^, duloxetina, venlafaxina
Opioides	Naltrexona
Toxina botulínica	Onabotulinum toxina A^1^, abobotulinum toxina A^1^, incobotulinum toxina A^1^, rimabotulinum toxina B^1^
Neuralgia del trigémino	
Línea de tratamiento	Tratamiento
Primera línea	Carbamazepina^1^, oxcarbazepina, acetato de eslicarbazepina
Segunda línea	Lamotrigina, baclofeno, gabapentina, pregabalina
Otros	Fenitoína, lidocaína, pimozida, tocaínida, clonazepam, topiramato, ácido valproico, levetiracetam, lacosamida

^1^ Evidencia clase 1 (evidencia respaldada por ensayos clínicos controlados) [22, 23]. Los medicamentos con un nivel de evidencia clase 1 se obtuvieron para las formas clásicas o idiopáticas de neuralgia del trigémino y dolor neuropático, pero en las formas secundarias, incluída la EM, la evidencia disponible es mayormente de estudios observacionales o series de casos, con nivel de evidencia por tanto, inferior.

La neuralgia del trigémino en EM es mucho más prevalente y resistente que en la población general. El tratamiento farmacológico es la primera opción, pero en casos con dolor resistente a medicamentos o mala tolerancia, se debe considerar la cirugía estereotáctica, o técnicas de neuromodulación cerebral o ultrasonido focalizado de alta intensidad (HIFU) y, si no mejora, considerar la cirugía percutánea [23].

### 4.2 Alteraciones Vesicales 

El manejo de las alteraciones vesicales tiene como objetivo prevenir las infecciones y daños del tracto urinario y mejorar la calidad de vida (CdV) del paciente [24]. Estos síntomas pueden evaluarse con un diario miccional y con cuestionarios validados (Tabla 2). Para los problemas de vaciado se recomiendan los alfa bloqueantes y el sondaje intermitente. Para la vejiga hiperactiva se recomendó implementar medidas comportamentales (evitar café y té), entrenamiento de suelo pélvico y tratamiento con adrenérgico β3 y estimulación tibial. Si estas medidas no funcionan, se puede recurrir a la neuromodulación sacral o toxina botulínica [24].

**Tabla 2.** **Cuestionarios validados**.

Síntoma o dominio evaluado	Cuestionario
Síntomas vesicales	Urinary Symptom Profile (USP)
	OverActive Bladder Symptom Score (OABSS)
	Neurogenic Bladder Symptom Score (NBSS)
Síntomas intestinales	Neurogenic Bowel Disorders questionnaire (NBD)
	Constipation Score System (CSS)
Disfunción sexual	Arizona Sexual Experience Scale (A-SEX)
	Multiple Sclerosis Intimacy and Sexuality Questionnaire (MSISQ15-19)

A-SEX, escala de experiencia sexual de Arizona; CSS, sistema de puntuación para el estreñimiento; MSISQ15-19, cuestionario de intimidad y sexualidad en la esclerosis múltiple; NBD, cuestionario de trastornos neurogénicos del intestino; NBSS, cuestionario de síntomas de vejiga neurógena; OABSS, cuestionario de síntomas de vejiga hiperactiva; USP, perfil de síntomas urinario.

### 4.3 Alteraciones del Equilibrio y la Marcha

Alrededor de 60–80% de las personas con EM presentan alteraciones del equilibrio, dando lugar a menor movilidad y mayor riesgo de caídas. Se ha visto que el protocolo tecnológico de rehabilitación con una plataforma robótica computerizada no es inferior al entrenamiento de equilibrio tradicional, mejorando el equilibrio y la marcha [25]. Por otra parte, un estudio de vida real indicó que el 60% de los pacientes tratados con fampridina mejoraron la marcha. La edad basal, el género, la puntuación basal en el escala de marcha en la EM (MSWS) y la escala expandida del estado de discapacidad (EDSS) predijeron la respuesta [26].

## 5. Rehabilitación Física y Cognitiva

### 5.1 Ejercicio Físico

**Fig. 2.**
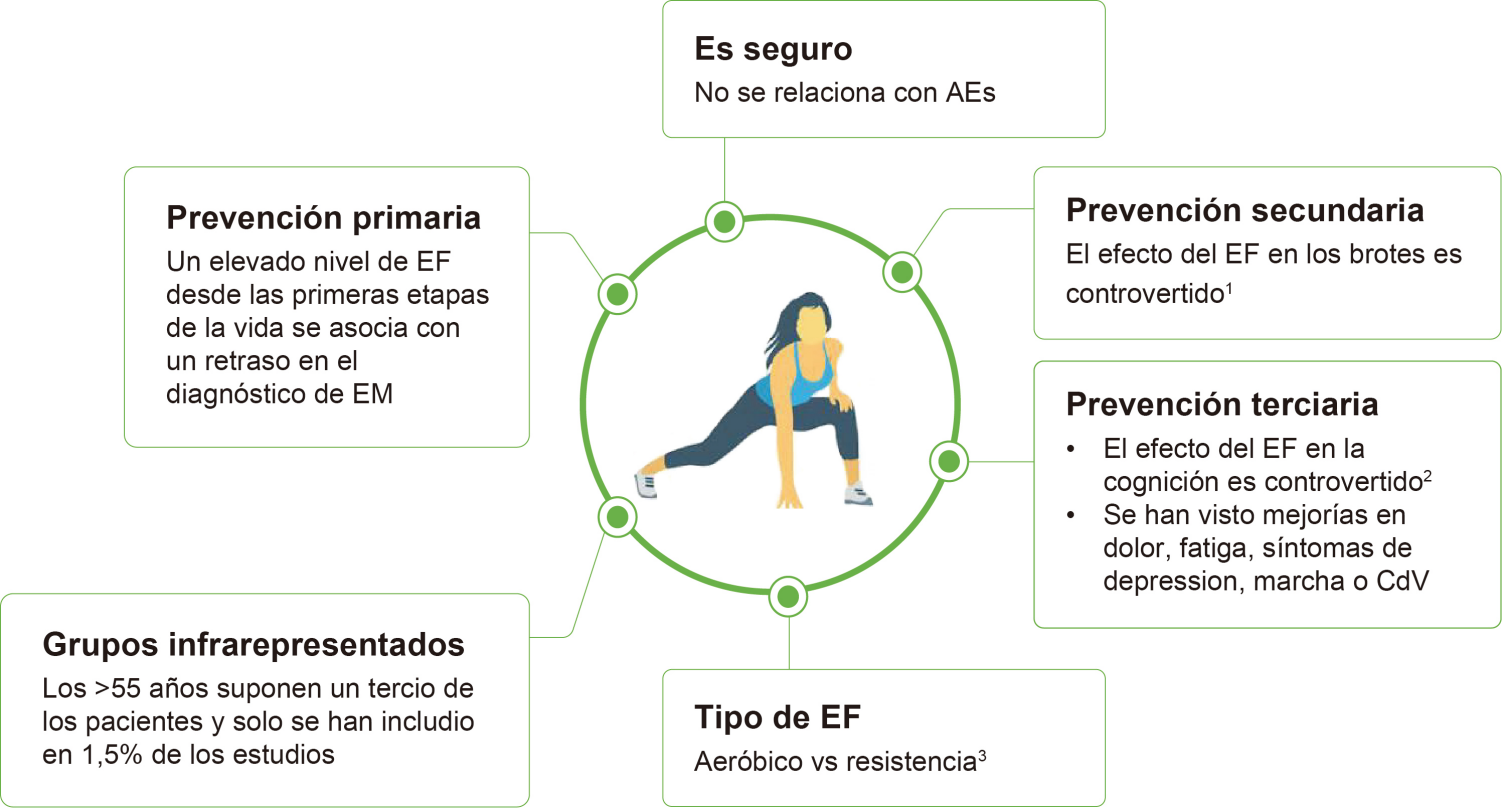
**Efecto del ejercicio físico sobre la EM**. AEs, acontecimientos adversos; CdV, calidad de vida; EF, ejercicio físico; EM, esclerosis multiple. ^1^Algunos estudios han demostrado un beneficio del EF en la aparición de los brotes, mientras que otros estudios no han observado dicho beneficio. ^2^Algunos meta-análisis han concluido que el EF mejora la función cognitiva en pacientes con EM [29], mientras que otros no han observado este efecto [28]. Estas diferencias en los resultados pueden ser debidas a varios problemas metodológicos tales como: (a) inclusión de EF de baja intensidad, (b) programa de EF no supervisado, (c) herramientas inapropiadas para evaluar la condición física, (d) estudio de un único dominio cognitivo, (e) programas de EF de corta duración (≤12 semanas), (f) resultados no ajustados en función del resto de covariables, (g) inclusión de pacientes sin afectación cognitiva basal, (h) inclusión de grupo control que no es totalmente inactivo. ^3^Los resultados del ensayo clínico MS BOOSTER, en el que los pacientes fueron aleatorizados a 12 semanas de entrenamiento de resistencia + cuidado estándar, entrenamiento aeróbico + cuidado estándar o solo cuidado estándar, mostraron que sólo el entrenamiento aeróbico mejoró la fatiga [30]. Esta figura fue creada utilizando la versión oficial de Adobe Illustrator.

El ejercicio físico (EF) puede proporcionar beneficios complementarios a los fármacos [27]. Los mayores beneficios del EF se han observado en la prevención primaria de la EM, mientras que su efecto en la prevención secundaria o terciaria es más dudoso (Fig. 2, Ref. [28, 29, 30]). La ausencia de efecto del EF en la cognición en EM reportada en varios estudios [28, 31] podría explicarse por limitaciones metodológicas y un enfoque equivocado en su evaluación. En lugar de esperar mejoras cognitivas tras unas semanas de intervención, cabría esperar que el EF contribuya a preservar la función cognitiva tras años de práctica constante [27].

Un ensayo clínico controlado (ECC) realizado en personas mayores de 60 años (PoTOMS) de 24 semanas de duración [32] mostró una mejoría significativa en el grupo que realizó EF en fuerza en manos y piernas y marcha, evaluado con la prueba de la marcha de 25 pies (T25FW) y la prueba de la marcha de los 2 minutos (2MWT), pero no se comprobó efecto alguno sobre la cognición, evaluado con la prueba de recordatorio selectivo (SRT9) y la prueba de modalidades símbolo-dígito (SDMT).

### 5.2 Reserva Cognitiva

El concepto tradicional de reserva es estático, siendo los factores que determinan la reserva no modificables [33]. El nuevo modelo de reserva que se propone es dinámico y vendría determinado por factores genéticos y ambientales (como en el modelo tradicional), pero además estaría influenciado por factores modificables. El mantenimiento de esta reserva dependería del equilibrio entre factores de riesgo (e.g., carga de la enfermedad, comorbilidades o dieta poco saluable) y protectores (e.g., efectividad de los TME y estilo de vida saludable). La pérdida de la reserva dependería de factores de riesgo (e.g., depresión, estrés, sedentarismo, y obsesidad) y de compensación (e.g., rehabilitación cognitiva) [34, 35].

En la ceremonia de apertura del ECTRIMS se comentó que debemos situar al paciente como eje de nuestra investigación y práctica clínica. Por otra parte, los pacientes con EM son expertos en detectar cambios en su función cognitiva. Si un paciente con EM dice que tiene un problema cognitivo, es probable que tenga un problema cognitivo. El trabajo del neurólogo consiste, por tanto, en averiguar la causa de esta alteración subjetiva y mejorar las exploraciones o su interpretación para detectar el deterioro cognitivo en la práctica clínica.

## 6. Fatiga y Sueño

### 6.1 Distinguiendo Fatiga y Depresión

La fatiga y la depresión son síntomas muy frecuentes en EM y con un alto impacto sobre la vida del paciente [36]. Un 37% y un 16% de los pacientes con EM reportaron que la fatiga y depresión, respectivamente, son muy graves o incapacitantes. Sin embargo, aún hay controversia sobre si la depresión es un síntoma central en la EM [37, 38].

Resulta difícil distinguir depresión y fatiga, ya que muchos de los síntomas se solapan. Se proponen diferentes fenotipos según combinación de síntomas (fatiga; fatiga y somnolencia; fatiga y depresión; fatiga, somnolencia y depresión) [39] o según magnitud de la fatiga (leve, leve-moderado; moderado-severo; severo; dominado por la fatiga; dominado por la salud mental) [40]. Se recomienda realizar una evaluación holística e intervenciones dirigidas al conjunto de síntomas [36].

### 6.2 Trastornos del Sueño

**Fig. 3.**
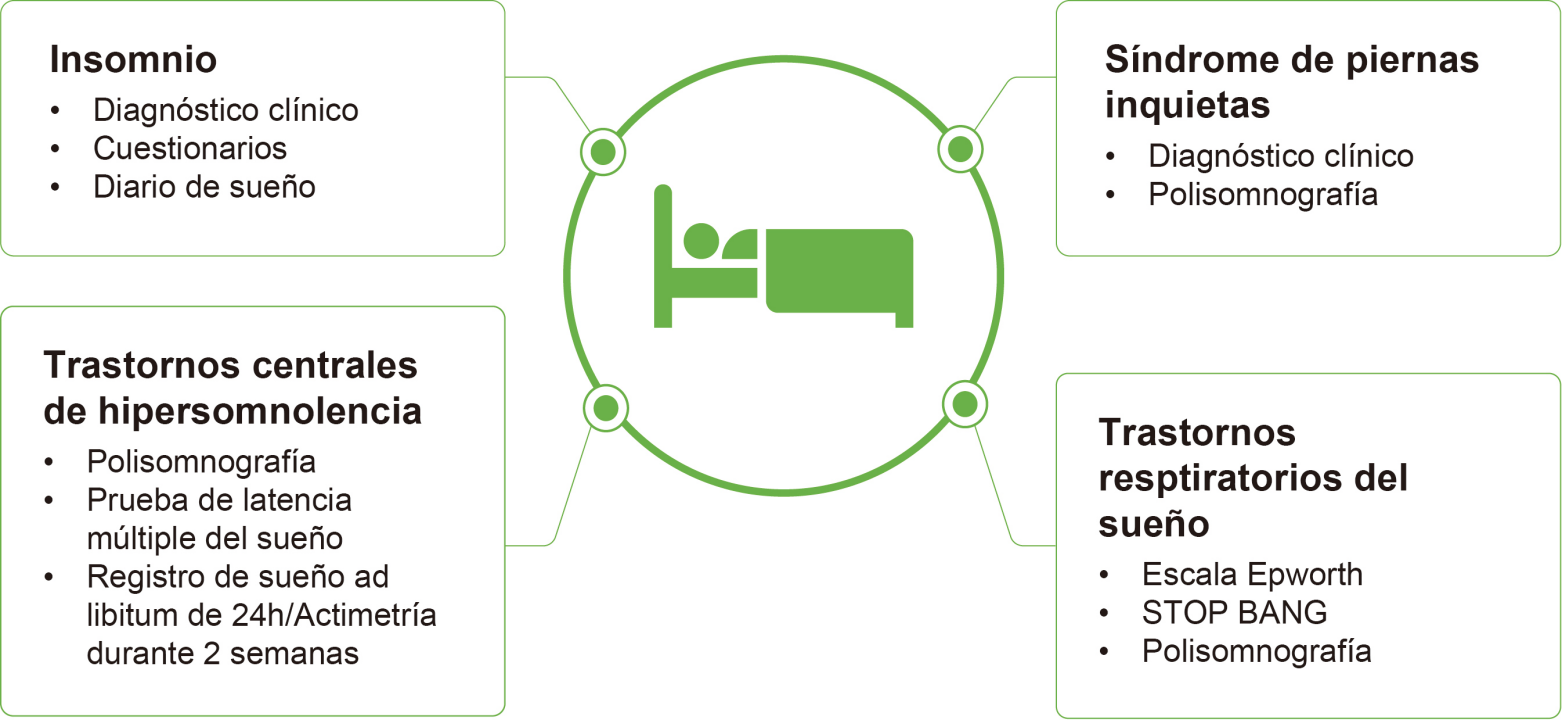
**Evaluación de los trastornos de sueño**. STOP-BANG es un cuestionario validado para la detección del riesgo de apnea obstructiva del sueño. Esta figura fue creada utilizando la versión oficial de Adobe Illustrator.

Más del 60% de los pacientes con EM presentan trastornos del sueño, pero menos de la mitad lo reportan. El tratamiento de los trastornos del sueño puede mejorar la fatiga [41]. Algunos de los trastornos del sueño más comunes en la EM, y más frecuentes que en la población general, son el insomnio, el síndrome de piernas inquietas, los trastornos respiratorios relacionados con el sueño y los trastornos centrales de hipersomnolencia [42]. La Fig. 3 muestra herramientas diagnósticas para identificar estos trastornos.

### 6.3 Fatiga y Neuroimagen

La fatiga se relaciona con una disfunción en los circuitos cortico-subcorticales. Los estudios de neuroimagen estructural muestran que, cuando se comparan personas con EM con y sin fatiga, no hay diferencias en la sustancia blanca de forma global, pero sí las hay en el lóbulo frontal y parietal y la cápsula interna. Lo mismo ocurre con la sustancia blanca de aspecto normal y en la atrofia de la sustancia gris [43]. Los estudios en lesiones corticales de la sustancia gris no son concluyentes, posiblemente debido a que no han analizado zonas concretas. En cuando a neuroimagen funcional, la fatiga está asociada con alteraciones específicas en las redes monoaminérgicas [44, 45]. El volumen del plexo coroideo parece ser el único predictor de la fatiga [46].

## 7. Desafíos en el Tratamiento de la EM

**Fig. 4.**
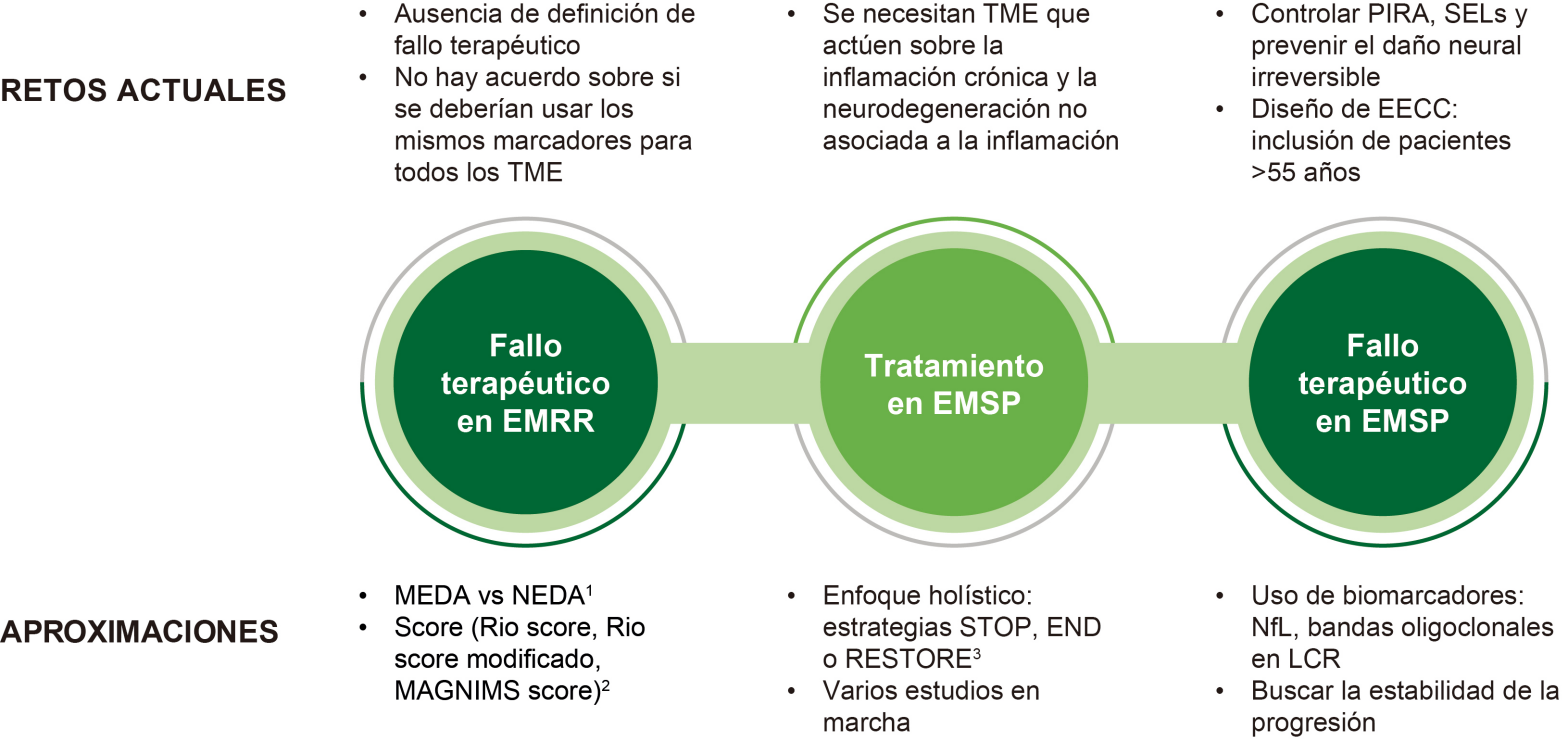
**Definición de fallo de tratamiento**. EECC, ensayo clínico; EMRR, esclerosis múltiple recurrente-remitente; EMSP, esclerosis múltiple secundaria progresiva; LCR, líquido cefalorraquídeo; MAGNIMS, red europea para el estudio de resonancia magnética en EM; MEDA, evidencia mínima de actividad de la enfermedad; NEDA, sin evidencia de actividad de la enfermedad; NfL, neurofilamentos; PRL, lesión con borde paramagnético; PIRA, progresión independiente de los brotes; SEL, lesión de expansión lenta; TME, tratamiento modificador de la enfermedad. ^1^La ausencia de NEDA-3 no necesariamente va asociado a una acumulación de discapacidad a largo plazo e, además, es una medida poco realista. En los primeros años del tratamiento podemos tolerar una actividad en RM mínima (<3 lesiones T2) sin exponer a los pacientes a riesgo de discapacidad futura (MEDA). ^2^Limitaciones: no tienen en cuenta la la localización de lesiones, ni evalúan cognición, medidas PROMS y más sensibles, neurofilamentos SEL o PRLs. ^3^Estrategia STOP: TME que abordan tanto procesos específicos de la EM como no específicos (promoción de la salud cerebral); estrategia RESTORE: tratamientos de remielinización y neuroprotecció; estrategias END: Tratamientos y enfoques preventivos. Esta figura fue creada utilizando la versión oficial de Adobe Illustrator.

La Fig. 4 muestra algunas de las reflexiones que se hicieron en ECTRIMS sobre los retos que siguen existiendo en la medición del fallo terapéutico y tratamientos para la EMSP [47, 48, 49].

## 8. Tratamiento de la EM: Datos de Vida Real

### 8.1 Efectividad

Cuando se va a elegir un TME, cabría preguntarse si se debe tomar esta decisión en base a la eficacia o a la seguridad, vía de administración o impacto en la CdV [50].

Los datos del registro sueco mostraron que en los pacientes tratados con rituximab, que representan el 62% del total de pacientes con EM, la progresión independiente de brotes (PIRA) es siete veces más común que el empeoramiento asociado a brotes (RAW). En estos pacientes, además, el PIRA era más prevalente cuando la EDSS ≥2 y presentaban más de 20 lesiones en RM [51].

### 8.2 Seguridad

Los acontecimientos adversos (AA) secundarios a fármacos representan la quinta causa de muerte en la población general en Europa, siendo la mitad de ellos prevenibles [52]. A pesar de la relevancia clínica de los AA, los ECC se siguen diseñando para evaluar eficacia. No obstante, la comunidad científica se esfuerza en recabar información sobre los AA (e.g., programa de leucoencefalopatía multifocal progresiva con natalizumab o de neoplasias con ocrelizumab).

Un estudio longitudinal con 25.000 pacientes mostró un riesgo significativamente mayor de hospitalización relacionada con infecciones en pacientes tratados con ocrelizumab [53]. Sin embargo, el estudio no reportó datos de discapacidad ni del tipo de EM, y el tamaño de la muestra de algunos de los TME era muy reducido, por lo que no se pueden extraer conclusiones de todos los tratamientos.

**Fig. 5.**
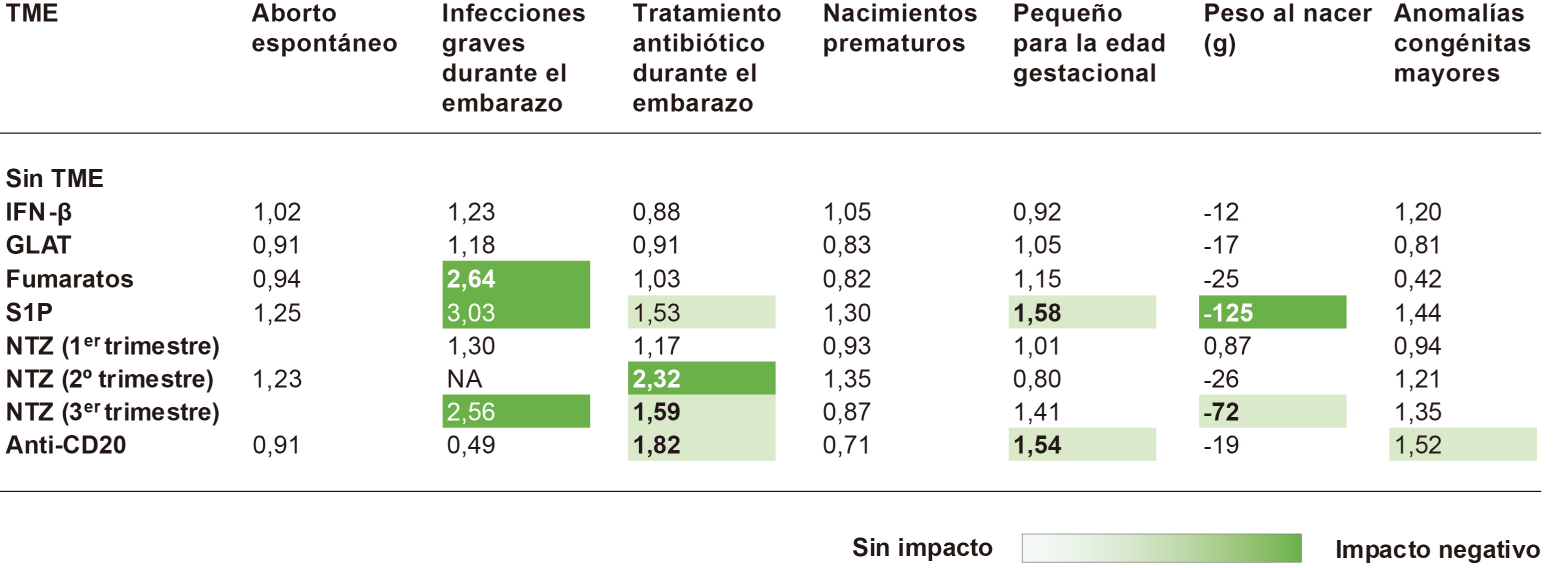
**Embarazo y resultados neonatales**. GLAT, glatiramero; IFN-β, interferón-beta; NTZ, natalizumab; S1P, moduladores del receptor de esfingosina-1-fosfato; TME, tratamientos modificadores de la enfermedad. La figura presenta un análisis de regresión sobre resultados del embarazo y neonatales en pacientes con EM expuestas a diferentes TMEs. Los datos se preentan como odds ratios (cuando la variable de resultado es dicotómica, i.e., todas las variables, excepto peso al nacer) y coeficientes β (cuando la variable de resultado es continua, i.e., peso al nacer). Los valores en negrita indican *p* < 0,05. Esta figura fue creada utilizando la versión oficial de Adobe Illustrator.

Se presentaron también los hallazgos del registro alemán de embarazos (2885 expuestos a TME según ficha técnica y 837 no expuestos). Todos los TME, excepto glatiramero e interferones, mostraron un aumento en los riesgos en mayor o menor medida (ver Fig. 5). Se observó un mayor número de recién nacidos pequeños para la edad gestacional en el grupo tratado, especialmente en madres tratadas con TME de alta eficacia. Los datos de alemtuzumab, teriflunomida y cladribina no se analizaron debido al bajo número de casos [54].

### 8.3 Inercia Terapéutica en Mujeres

Con el objetivo de investigar si la inercia terapéutica afecta más a las mujeres en edad fértil con EM, Gavoille [55] analizó a las pacientes entre 18 y 40 años utilizando una base de datos desde 1997 a 2022 (N = 22,657; 74% mujeres). Los resultados mostraron que las mujeres estaban menos tratadas que los hombres, en general, y con TME de alta eficacia en particular (*p* < 0,001). Además, el uso de TME y TME de alta eficacia fue menor los tres años antes del embarazo, disminuyendo considerablemente sobre los 9 meses antes de la concepción.

Probablemente, gracias a los datos de seguridad recogidos en los últimos años, esta inercia terapéutica se verá disminuida y se tratará en mayor medida a las mujeres que precisen un TME, incluyendo los de mayor eficacia.

### 8.4 Ensayos Aleatorizados Basados en un Registro 

Los ensayos aleatorizados basados en un registro son una alternativa para generar evidencia más representativa y aplicable a la práctica clínica que la proporcionada por los ECC. Estos estudios combinan las características de los ECC con los registros de datos de la vida real. Los pacientes se asignan aleatoriamente a diferentes tratamientos, como en un ECC, pero el seguimiento y la recopilación de datos se realizan a través de bases de datos existentes [50]. Las ventajas de este tipo de estudios son una mayor eficiencia en coste y tiempo, relevancia clínica y mayor representatividad y generalización de las conclusiones.

## 9. Lo Más Destacado Sobre Cladribina

La Tabla 3 (Ref. [56, 57, 58, 59, 60, 61, 62, 63, 64]) resume las comunicaciones más destacadas sobre el efecto de cladribina en el control sostenido de la EM y la confirmación de su seguridad [56, 57, 58, 59, 60, 61]. Los estudios muestran un perfil de seguridad favorable a largo plazo y en personas mayores de 50 años [2, 65, 66]. De hecho, la tasa de AA de especial interés disminuye progresivamente a lo largo del tiempo [67].

**Tabla 3.** **Resumen de los nuevos datos de cladribina presentados en ECTRIMS 2024**.

Estudio [ref]	Periodo de observación; N	Resultados de efectividad	Resultados de seguridad
Cohorte de Madrid [56]	4 años; N = 552	• El 83% de los pacientes permaneció libre de brotes	• No se observaron AAs más allá de los ya notificados en los ECC
		• La probabilidad de estar sin PCD alcanzó el 87%	
		• El 49,3% de los pacientes se mantuvo en NEDA	
		• El porcentaje de pacientes libre de brotes fue similar en pacientes naive y previamente tratados con tratamientos plataforma	
Cohorte de Madrid [62]	4 años; N = 145	• Cladribina redujo de forma significativa los neurofilamentos a lo largo del período de observación; sin embargo, al final del cuarto año se observa un repunte. Este aumento plantea que los pacientes en los que se detecte dicha elevación al final del cuarto año podrían beneficiarse de la continuación del tratamiento con cladribina.	• No reportado
Cohorte de Suecia [57]	4 años; N = 369	• Reducción significativa de la TAB, desde basal (0,29) hasta el año 1 (0,06) hasta el año 4 (0,02)	• El perfil de seguridad fue coherente con lo observado durante los ECC y la experiencia post-comercialización
		• Reducción significativa en la frecuencia de lesiones. El 90,6% y 95,7% de los pacientes estaban libres de lesiones en el año 1 y 4, respectivamente	
		• La EDSS se mantuvo	
		• Se observa mejoría en la CdV (MSIS-29) y la gravedad de la enfermedad (MSSS)	
MAGNIFY-MS Extensión [58]	4 años; N = 219	• El 78,6% y el 79,2% de los pacientes se mantuvieron en NEDA en el año 3 y 4, respectivamente	• No se observaron nuevas señales de seguridad
MSBase [59]	4 años; N = 206	• A los 4 años, el 80% de los pacientes estaba libre de brotes y el 90% estaba sin PCD	• No reportado
		• Anualmente, >95% de los pacientes continuaba en tratamiento con CLAD y no requirieron cambio a otro TME	
Cohorte gallega CLADRIGAL [60]	2 años; N = 48	• Reducción significativa de la TAB desde basal (0,91) hasta el año 1 (0,13) y año 2 (0,14)	• CLAD fue bien tolerado por la mayoría de los pacientes, con un perfil de seguridad comparable a la cohorte total (≥50 y <50 años)
	pacientes mayores de 50 años.	• Reducción significativa en la tasa de lesiones desde la basal (0,58) hasta el Año 1 (0,05) y Año 2 (0,00)	
		• La discapacidad se mantuvo estable	
Cohorte de Sarasota [61]	4 años; N = 40	• Todos los pacientes permanecieron libres de brotes del año 2 al año 5	• No reportado
	pacientes mayores de 50 años.	• En el año 1 y 4, todos los pacientes permanecieron estables o mejoraron la actividad en RM (4 y 5 pacientes con dato disponible, respectivamente)	
[63]	2 años; N = 52	• Reducción significativa en el número de lesiones con anillo paramagnético a los 2 años	• No reportado
[64]	2 años; 359 (CLARIFY-MS)+ 256 (MAGNIFY-MS)	• Reducción en las SELs y las lesiones activas	• No reportado

AA, acontecimientos adversos; ECC, ensayo clínico controlado; EDSS, escala expandida del estado de discapacidad (*Expanded Disability Status Scale*); CLAD, cladribina cápsulas; MFIS-29, *Multiple Sclerosis Impact Scale*-29; MSSS, *Multiple Sclerosis Severity Score*; NEDA, ausencia de evidencia de actividad de la enfermedad (*no evidence of disease activity*); PCD, progresión confirmada de la discapacidad; RM, resonancia magnética; SELs, lesiones de expansión lenta (*Slowly Expanding Lesions*); TAB, tasa anualizada de brotes.

Respecto al impacto de cladribina en el sistema nervioso central (SNC), se ha visto que disminuyen los linfocitos B de memoria y CD27^+^ en el SNC, así como las citoquinas relacionadas con activación de microglía en el LCR [68, 69, 70], probablemente gracias a que el 25% de cladribina atraviesa la barrera hematoencefálica [71].

## 10. Terapias de Reconstitución Inmune

**Fig. 6.**
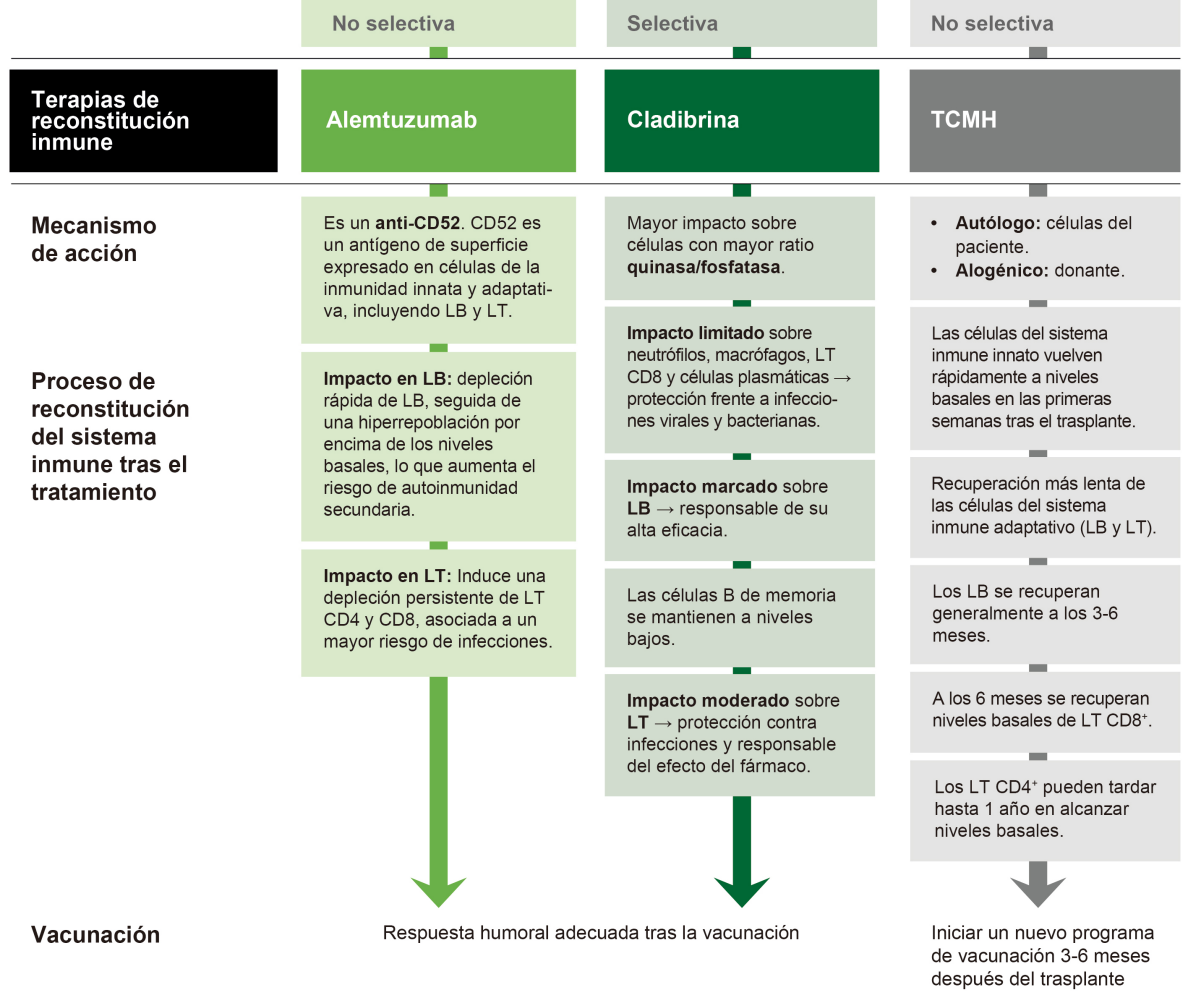
**Terapias de reconstitución immune**. LB, linfocitos B; LT, linfocitos T; TCMH, trasplante de células madre hematopoyética. Esta figura fue creada utilizando la versión oficial de Adobe Illustrator.

La Fig. 6 ilustra el mecanismo de acción y el proceso de reconstitución del sistema inmune tras el tratamiento con terapias de reconstitución inmune.

## 11. El futuro de las Nuevas Terapias en la EM

Los primeros resultados del uso de células CAR-T dirigidas a CD19 en dos pacientes con EM progresiva han mostrado un perfil de seguridad aceptable. Se detectaron células CAR-T en el LCR sin signos de neurotoxicidad, y una reducción de anticuerpos intratecales, sugiriendo un efecto directo sobre las células CD19^+^ [72]. Actualmente, hay cuatro estudios en marcha que evalúan terapias de células CAR-T en EM [73]. El tratamiento con células CAR-T podría ser una opción prometedora, aunque su elevado coste limitaría su accesibilidad.

Otra de las terapias más novedosas es la vía de coestimulación CD40–CD40L, la cual regula las respuestas inmunitarias adaptativas e innatas [74]. Un ensayo fase 2 ha mostrado que la inhibición de CD40L con frexalimab produce una mayor reducción en el número de nuevas lesiones captantes de gadolinio en T1 a la semana 12 en comparación con el placebo [74]. La extensión abierta del estudio ha mostrado que, al menos hasta la semana 72, hubo una reducción sostenida de la actividad de la enfermedad (clínica y radiológica), una estabilidad de la discapacidad (EDSS), los recuentos de linfocitos permanecieron estables y frexalimab fue bien tolerado [75].

Por último, los resultados a largo plazo del estudio de fase 1 sobre el trasplante de células madre neurales en EM progresiva mostraron efectos neuroprotectores sostenidos [76]. Se presentaron también los resultados del ECC D-lay EM. El estudio concluyó que dosis altas de colecalciferol (100.000 UI quincenales) reducen la actividad de la enfermedad después de un síndrome clínicamente aislado, posicionándolo como un candidato para ser evaluado como terapia complementaria en la estrategia terapéutica para la EM [77].

## 12. Conclusiones

En el post-ECTRIMS de 2024 se presentaron varias novedades sobre investigación en EM. En el ámbito del embarazo, se comentó que era seguro utilizar técnicas de reproducción asistida en pacientes con EM. En mujeres ya embarazas, los nuevos datos sobre TME, entre ellos de cladribina, podrían contribuir a la reevaluación de las fichas técnicas. A día de hoy, la inercia terapéutica en mujeres en edad fértil es mayor, estando menos tratadas que los hombres, particularmente con TME de alta eficacia. Se resume el impacto de diferentes TME en el embarazo y los resultados neonatales.

Dados los cambios inmunológicos a lo largo de la vida, se subrayó la necesidad de utilizar estrategias personalizadas, especialmente en pacientes mayores, donde los riesgos de efectos adversos son mayores, la progresión se acelera y la respuesta a los TME cambia. Dentro de los retos que persisten, se resaltó la medición del fallo terapéutico y los TME para la EMSP. Se enfatizó la importancia de recopilar información de seguridad, y se propusieron los ensayos aleatorizados basados en registro como estrategia para generar evidencia más representativa y aplicable a la práctica clínica que los ECC.

Por otra parte, se recalcó que, si un paciente con EM indica que tiene un problema cognitivo, estas alteraciones deben evaluarse en consulta. También se destacó la importancia de preguntar activamente por la disfunción sexual y los trastornos de sueño en la consulta, dada su alta prevalencia en esta población.

Finalmente, se mostraron resultados preliminares positivos sobre terapias emergentes con células CAR-T y los inhibidores de CD40–CD40L, lo que representa un avance prometedor en el manejo de la EM.

## Contribuciones de los Autores

Todos los autores del Grupo Post-ECTRIMS han contribuido por igual en la elaboración de este artículo. OF, AArés, EA, YA, AAlonso, RA, LB, CC, ABC, TCT, LCF, SE, MAH, LL, ML, SL, JML, VML, EM, COG, JMP, LRP, AV, ARA han contribuciones sustanciales a la concepción o diseño de la obra, participado en la redacción del manuscrito o en su revisión crítica por contenido intelectual relevante, han dado su aprobación final a la versión que será publicada y han aceptado la responsabilidad por todos los aspectos del trabajo, asegurando que cualquier cuestión relacionada con la exactitud o integridad de alguna parte de este sea debidamente investigada y resuelta.

## Aprobación Ética y Consentimiento Informado

No aplicable.

## Agradecimientos

Los autores quisieran mostrar su agradecimiento a Laura Prieto del Val e Isabel Caballero de Evidenze Health España, S.L.U por los servicios de redacción médica prestados en la realización del artículo.

## Financiación

La elaboración de este artículo ha sido financiada por Merck.
